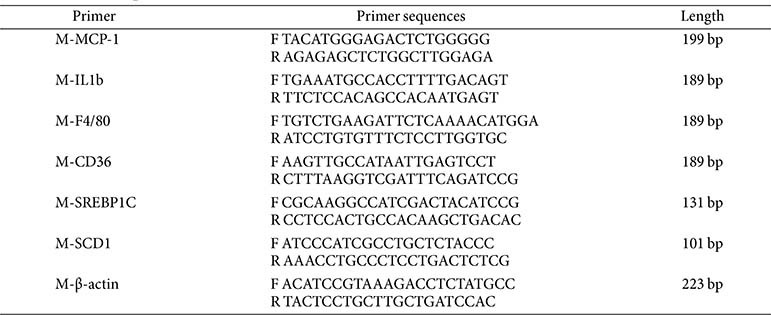# L-Theanine Ameliorates Metabolic Dysregulation and Adverse Fetal Outcomes in a Mice Model of Gestational Obesity: Association with FXR/FGF15 Signaling

**DOI:** 10.4014/jmb.2504.04017

**Published:** 2025-09-22

**Authors:** Le Huang, Hua Li, Weitao Yang, Lihui Huang, Qiuling Chen, Shengnan Li, Zhi Zou, Lijing Zhao, Zhihua Zeng

**Affiliations:** Department of Obstetrics, Changsha Hospital for Maternal & Child Health Care Affiliated to Hunan Normal University, Changsha 410007, P.R. China

**Keywords:** L-theanine, FXR/FGF15, gut microbiota, gestational obesity, fecal microbiota transplantation

## Abstract

In this study, we investigated whether L-theanine (LTA) ameliorates adverse pregnancy outcomes in high-fat diet (HFD)-induced gestational obesity mice. Gestational obese mice models received HFD and fecal microbiota transplantation (FMT) from pregnant obese women, followed by LTA treatment. Gut microbiota DNA from six obese and six normal pregnant women was analyzed. Also assessed were lipid profiles, inflammatory factors, gut permeability, FXR/FGF15 expression, pup weight, and placental function. Alpha- and beta-diversity analyses showed reduced gut microbial diversity in the obese pregnant women. Postpartum hemorrhage, cholesterol, and triglycerides inversely correlated with *Weissella*, while BMI was positively associated with *Escherichia-Shigella*. Neonatal weight correlated positively with *Subdoligranulum* and negatively with *Megamonas*. Fasting glucose was significantly positively associated with *Bacteroides vulgatus*, whereas neonatal body weight inversely correlated with *Eubacterium ramulus*. In gestational obesity mice, LTA administration reduced weight gain, visceral/gonadal adiposity, metabolic markers (fasting glucose/insulin/cholesterol), gut barrier dysfunction (TNF-α, IL-6, IL-8, Claudin-2), and linked to FXR/FGF15 pathway alterations. Furthermore, LTA intervention suppressed MCP-1, IL-1β, F4/80 and hepatic lipid metabolism regulators (CD36, SREBP1c, SCD1, GLUT4, Cyp7a1, IRS-1), while also mitigating placental tissue junction zone abnormalities and pup weight. To sum up, LTA-mediated attenuation of adverse pregnancy outcomes associates with FXR/FGF15 pathway alterations, concomitant with restoration of metabolic homeostasis and inflammation suppression.

## Introduction

Over the past decades, the global prevalence of high weight gain and obesity has reached alarming proportions, affecting approximately 40% of adults [1]. The incidence of neonatal obesity is also rising, with an escalating proportion of women of childbearing age being recorded as obese [2]. Globally, 20 40% of pregnant women are overweight or obese. Gestational obesity (GO), defined by excessive gestational weight gain and metabolic dysregulation, represents a predominant metabolic disorder during pregnancy and constitutes a major public health challenge worldwide [3]. GO exacerbates the risk of gestational diabetes mellitus (GDM), hypertension, and other complications, while also correlating strongly with adverse pregnancy outcomes, such as fetal growth restriction, preeclampsia, and intergenerational metabolic dysregulation [4]. Despite extensive investigations into the pathophysiology of GO, effective therapeutic interventions remain insufficient. Existing interventions, including dietary adjustments and physical activity regimens, frequently demonstrate inconsistent efficacy owing to low adherence and interindividual heterogeneity [5, 6]. Additionally, pharmacological interventions are constrained by their potential teratogenic risks, highlighting the demand for safer and more efficacious therapeutic alternatives [7]. Consequently, elucidating the underlying mechanisms of GO is imperative for mitigating adverse pregnancy outcomes and enhancing maternal-fetal health.

The high-fat diet (HFD) is recognized as a significant contributor to GO, primarily through modulating gut microbiota structure and function, which subsequently perturbs host metabolic homeostasis. Emerging evidence demonstrates that specific dietary supplements, including metformin [8], probiotics [9], and nicotinamide mononucleotide (NMN) [10], ameliorate fertility deficits and offspring metabolic health in HFD-induced obese murine models. Therefore, investigating novel dietary interventions could unveil innovative approaches to improve reproductive outcomes in GO models. Gut microbiota serves as a central nexus in diet-metabolic health research [11], mediating host interactions through metabolites, particularly via the bile acid metabolism pathway, which critically governs energy homeostasis and inflammatory regulation [12]. In addition, growing evidence implicates gut microbiota dysbiosis as a key driver in the development of GO [13]. As a microbiota-modulating intervention, fecal microbiota transplantation (FMT) has been extensively utilized in metabolic disease studies. Herein, we employed FMT to reconfigure the gut microbiota composition and functionality in GO mice, thereby evaluating its therapeutic potential in mitigating adverse pregnancy outcomes.

L-theanine (LTA), chemically known as γ-glutamylethylamide, is a naturally occurring, non-protein amino acid primarily found in tea. It exhibits multiple physiological functions, including antioxidant, anti-inflammatory, lipid-lowering, and immune-regulatory effects, and has a positive impact on cognitive ability, emotional state, sleep quality, and the management of metabolic diseases, such as obesity and cardiovascular diseases [14, 15]. Existing studies have demonstrated the beneficial effects of LTA in HFD-induced obesity models [16]. Furthermore, research by Peng Wanqiu *et al*. indicates that LTA ameliorates diet-induced obesity in mice by modulating the gut microbiota, thereby restoring intestinal barrier integrity and inducing white adipose tissue browning [17]. Given the well-established role of gut microbiota dysbiosis as a pivotal factor in the pathogenesis of obesity and related metabolic disorders [18], alongside the therapeutic potential of FMT [19], coupled with LTA's demonstrated ability to modulate microbial composition [20], elucidating the specific mechanisms of LTA within FMT+HFD-induced gestational obesity models represents a crucial research imperative.

Additionally, LTA can modulate glucose, lipid, and protein metabolism through insulin and adenosine 5‘-monophosphate-activated protein kinase (AMPK) and their downstream signaling pathways [17]. The farnesoid X receptor (FXR) is a key regulator of bile acid metabolism, while the downstream signaling molecule fibroblast growth factor 15 (FGF15) plays a crucial role in maintaining gut-liver axis homeostasis [18]. Research demonstrates that LTA regulates lipid metabolism in mice by modulating gut microbiota and bile acid metabolism via the FXR-FGF15-cholesterol 7 α-hydroxylase (CYP7A1) pathway [19]. However, the role of FXR in GO remains incompletely understood, presenting an opportunity to explore novel intervention strategies.

Given the context mentioned above, we established a GO mice model induced by the combination administration of HFD and FMT. We then systematically evaluated the impacts of LTA on maternal metabolism, fetal development, and the FXR/FGF15 signaling pathway by integrating gut microbiota analysis, metabolomics, and molecular biology techniques. This research not only provides novel insights into the mechanisms underlying GO, but also offers experimental evidence supporting the potential of LTA as an effective intervention strategy.

## Materials and Methods

### Patients and Sample Collection

Maternal clinical data on 28 pregnant women, encompassing pregnancy outcomes and neonatal characteristics, were retrieved from the outpatient electronic medical record system. Women with obesity during pregnancy were enrolled based on a BMI ≥25 kg/m^2^ measured at 12-20 gestational weeks [20]. The normal pregnancy group comprised women with a BMI of 18.5-24.9 kg/m^2^. All enrolled women were singleton pregnancies and abstained from antibiotic use throughout pregnancy. The cohort consisted of 14 individuals per group, with a total sample size of 28. Clinical information for the two groups of pregnant women was presented in Table S1. Fecal samples obtained during delivery were subjected to 16S rRNA gene sequencing. Written informed consent was obtained from all participants, and the study was approved by the Institutional Review Board of Changsha Hospital for Maternal & Child Health Care (Approval No. EC-20240321-02).

### Animals and Groups

Seven-week-old C57BL/6J mice (16-20 g) were purchased from Tianqin Biotechnology Co. Ltd., (China) and acclimatized for one week under conditions of 20-24°C, 40-50% relative humidity, and a 12/12-h light/dark cycle. Female and male mice were co-housed in a 2:1 ratio. The vagina was examined the following day, with the presence of sperm or a vaginal plug indicating day 0 of pregnancy (D0). LTA powder (200 mg; 3081-61-6, Chengdu Herpurify, China) was dissolved in 10 ml of saline to make a solution with a final concentration of 20 mg/ml.

Fecal microbiota treatment: Fecal samples from 40-week pregnant women were collected over at least 7 consecutive days. The fecal samples were filtered to collect fecal microbiota fluid, which was then centrifuged at over 4,000 ×*g* to obtain the fecal microbiota. Pregnant mice were randomly assigned to four groups under SPF conditions: the normal diet group (NDG) mice had a normal diet (10% fat) +15 mg gavage of feces from normally pregnant women. The HFD group (HFDG) mice were given an HFD (45% fat, Jiangsu Xie Tong Bio) +15 mg gavage of feces from obese pregnant women. In the NDG+LTA group, mice had a normal diet +15 mg gavage of feces from normally pregnant women +200 mg/kg LTA gavage. The HFDG+LTA group of mice was given an HFD+15 mg gavage of feces from obese pregnant women +200 mg/kg LTA gavage [21]. For LTA intervention, LTA was administered orally via gavage at 200 mg/kg, with continuous intervention until delivery. For all experimental mice groups, the following parameters were systematically documented: parturition timing, litter size and pup weight, placental weight, and visceral/gonadal adipose tissue mass. At the experimental endpoint, all animals were euthanized via cervical dislocation under anesthesia induced by 5% isoflurane. Death was confirmed by cessation of respiration and heartbeat, along with pupil dilation. All procedures were approved by the Ethics Committee of Hunan Normal University (Approval ID: No. 2024-693) and conducted in compliance with ARRIVE guidelines.

### 16S rRNA Gene Sequencing and Metabolomics Analysis

Total microbial DNA was isolated from fecal samples, with purity confirmed by NanoDrop 2000 (A_260_/A_280_ ratio of 1.8–2.0) (Thermo Fisher Scientific, USA). Amplification of the V3–V4 region was performed with primers 341F/806R using a PCR protocol. Libraries were quality-assessed and subjected to paired-end sequencing on an Illumina NovaSeq 6000 platform (USA). Raw reads were processed with Trimmomatic v0.39 (Q30 threshold >80%), denoised using the DADA2 algorithm, and clustered into ASVs. Species annotation was conducted against the SILVA database (v138), while α diversity (Shannon and Chao1 indices) and β diversity (Bray-Curtis distance-based PCoA) were analyzed using QIIME2. Group-specific microbial biomarkers were determined through LEfSe analysis (LDA score >3.0, *p* < 0.05).

### Enzyme-Linked Immunosorbent Assay (ELISA)

The following kits were used to detect the indicators: IL-6 (CSB-E04639m, Cusabio), IL-8 (EM1592, FineTest), TNF-α (CSB-E04741m, Cusabio), FGF15 (CSB-EL522052MO, Huamei), INS (CSB-E05071m, Cusabio), total cholesterol (A111-1-1, Nanjing Jiancheng Bioengineering) and glucose (A154-1-1, Nanjing Jiancheng Bioengineering) all purchased from China. Blood samples were centrifuged at 1,000 ×*g* for 15 min at 2-8°C, and the supernatant was immediately collected for detection. The BCA Protein Assay Kit (CW0014S, CWBio, China) was used to prepare a standard curve for calculating protein concentrations. Standard and sample wells were established in a 96-well plate, with 100 μl of standard or sample added to each well and incubated at 37°C for 2 h. Biotin-labeled antibodies and horseradish peroxidase-labeled avidin working solution were added sequentially and incubated for 1 h. The wells were washed after discarding the liquid. Subsequently, 90 μl of substrate solution was added, and the plate was incubated at 37°C in the dark for 15-30 min for color development. Finally, 50 μl of stop solution was added to terminate the reaction, and the optical density (OD) value of each well was measured at 450 nm using a microplate reader (MB-530, Huisong, China) within 5 min.

### Western Blot (WB)

For protein extraction, 300 μl of RIPA lysis buffer (AWB0136, Abiowell, China) was added to the 0.025 g tissue sample. The protein concentration of the samples was determined using the BCA method. A 10% separating gel and a 4.8% stacking gel were prepared for protein electrophoresis blotting, which was conducted at 75V for 150 min. After transfer, the membrane was washed once with 1*PBST and then incubated with 5% skimmed milk at room temperature on a shaker for 90 min. The primary antibody was diluted according to the ratios shown in Table 1, and the membrane was incubated with the primary antibody overnight at 4°C. Subsequently, the membrane was incubated at room temperature for 90 min with goat anti-mouse IgG (H+L) secondary antibody, HRP (1:5000, AWS0001, Abiowell) and goat anti-rabbit IgG (H+L) secondary antibody, HRP (1:5000, AWS0002, Abiowell). Finally, the membrane was treated with ECL chemiluminescent solution for 1 min, and imaged using a gel imaging system (ChemiScope6100, Qinxing, China).

### Quantitative Real-Time PCR (qRT-PCR)

Total RNA was extracted from cells or 0.02 g of tissue using Trizol reagent (15596026, Thermo Fisher Scientific, USA), and the RNA concentration and purity were determined using a spectrophotometer with an A_260_/A_280_ ratio, and RNA integrity was assessed using agarose gel electrophoresis. Reverse transcription was performed using 7 μg of total RNA, with the reaction system prepared according to the reverse transcription kit's instructions. The reaction conditions were 25°C for 5 min, 42°C for 30 min, and 85°C for 5 min. A 20 μl reaction system was prepared as follows: 2 μl of DNA, 0.8 μl of forward primer (10 μM), 0.8 μl of reverse primer (10 μM), 10 μl of SYBR Green Master Mix, and ddH_2_O was added to a final volume of 20 μl. Three replicate wells were established for each sample. The reaction program was 95°C for 10 min, then 40 cycles of 95°C for 15 sec and 60°C for 30 sec, and melting curve analysis from 65°C to 95°C, with a temperature increase of 0.5°C every 5 sec. The relative expression levels of target genes were quantified using the 2^−ΔΔCt^ method, with β-actin as the reference gene, and statistical analyses were conducted using GraphPad Prism software. The primer sequencer was shown in Table 2.

### Hematoxylin and Eosin (HE) Staining

The sections were baked at 60°C for 2-3 h and subsequently underwent deparaffinization. The sections were then sequentially incubated in 100%, 95%, 85%, and 75% ethanol (W990001-1, Tianjin Zhiyuan Chemical Reagent, China) for 5 min per gradient, with a final 5-min wash in distilled water. Following that, the sections were stained with hematoxylin for 1–10 min, rinsed with distilled water, counterstained with PBS for bluing, and then stained with eosin for 1–5 min, followed by another distilled water rinse. Gradient dehydration was performed using 95%–100% ethanol (5 min per step), or the sections were oven-dried. After a 10-min immersion in xylene, the sections were mounted with neutral resin and examined under a microscope.

### Immunofluorescence (IF) Staining

The sections were baked at 60°C for 12 h and then subjected to deparaffinization in xylene. Subsequently, they were immersed in 0.01 M citrate-based antigen retrieval buffer and heated to boiling in a microwave oven (MM721AAU-PW, Midea). After the power was turned off, the sections were allowed to cool and then equilibrated to room temperature. The sections were then rinsed with 0.01 M phosphate-buffered saline (PBS), incubated in sodium borohydride solution at room temperature for 30 min, washed with water for 5 min, treated with 75% ethanol for 1-5 min, stained with Sudan Black for 15 min at room temperature, and finally rinsed with water for 3 min. The sections were then blocked with a solution containing 10% normal serum and 5% BSA (AWI0120a, Abiowell). Following incubation overnight at 4°C with the diluted Claudin-2 primary antibody (AWS0005a, 1:200, Abiowell), the sections were incubated with anti-rabbit IgG fluorescently labeled antibody (50–100 μl) at 37°C for 60 min. Nuclei were counterstained with DAPI working solution at 37°C for 10 min, washed with PBS for 5 min, mounted with buffered glycerol, and examined under a fluorescence microscope (BA410T, Motic, China).

### Alcian Blue Staining

The Alcian Blue staining kit (AWI0607a, Abiowell) was utilized for analysis. The sections were baked at 60°C for 30-60 min. For deparaffinization, sections were immersed in xylene (10023418, Sinopharm, China) for 20 min. The sections were dehydrated through a graded ethanol series (100%, 95%, 85%, 75%) for 5 min per step. The sections were then stained with Alcian Blue solution for 5 min at room temperature, washed under running water, and rinsed with distilled water. Counterstaining with Nuclear Fast Red was then conducted, and the sections were rinsed with distilled water. After drying at 60°C for 30-60 min, sections were cleared in xylene (two changes, 10 min each), mounted with neutral resin, and microscopically examined.

### Statistical Analysis

Statistical analyses were performed using GraphPad Prism 8.0 software. Data for normally distributed samples are presented as the mean ± SD. Comparisons between two groups were conducted using Student's *t*-test, while one-way ANOVA was applied for comparisons across multiple groups. Correlation analyses at the phylum or genus levels were performed using Spearman's rank correlation test. Tukey's post hoc test was applied for multiple comparisons. Statistical significance was defined as a *p*-value < 0.05. All experiments were replicated at least three times.

## Results

### Reduced Gut Microbiota Species Richness in Women with GO

**Fig. 1:**
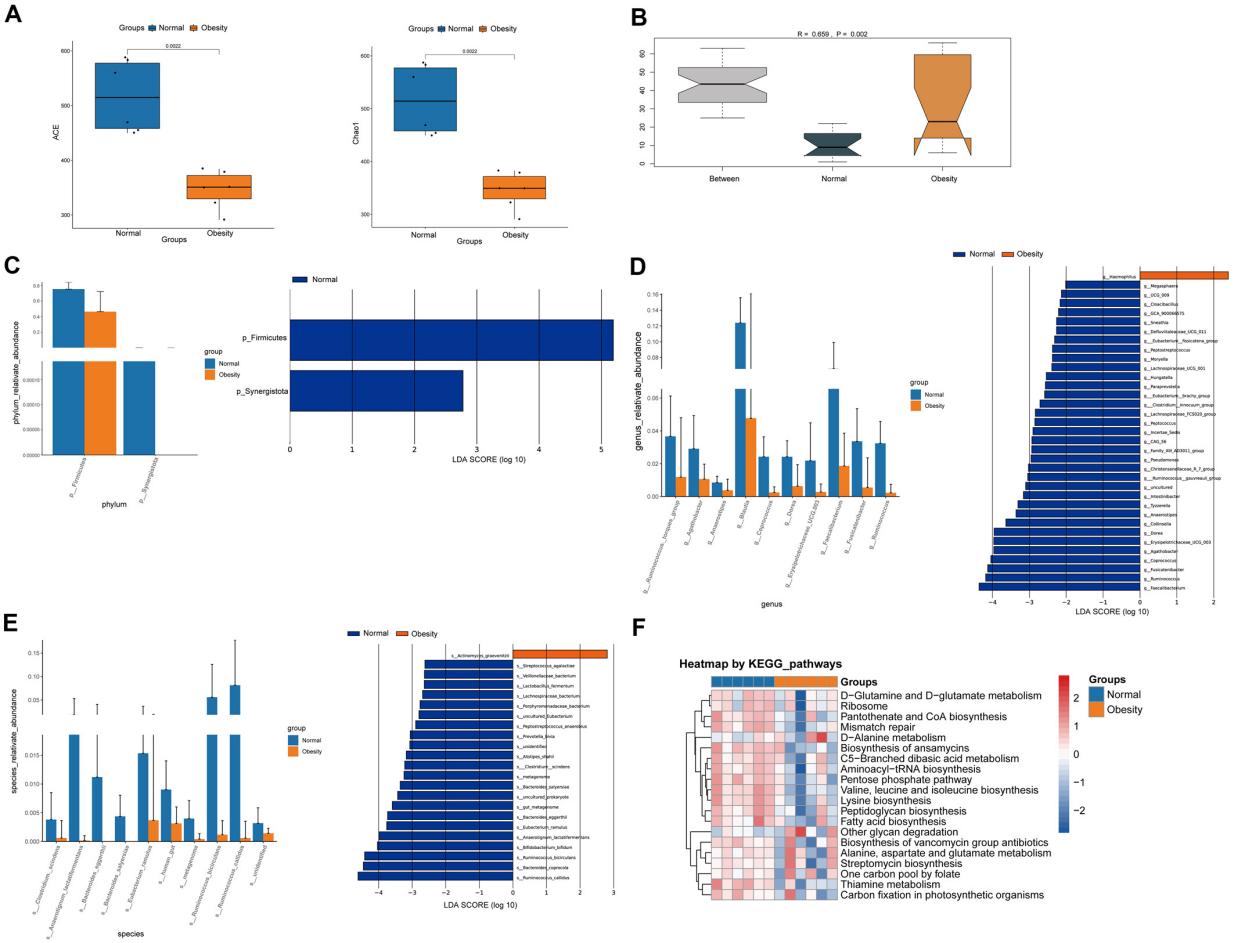
Reduced gut microbiota species richness in women with GO. (**A**) α-diversity analysis of gut microbiota in normal and obesity pregnancy groups. The figure shows three indicators: ACE (left) and Chao1 (middle). The normal group exhibited significantly higher ACE and Chao1 values than the obesity group (*p* = 0.0022), indicating greater species richness in the normal group. (**B**) Simpson index distribution analysis revealed significant intergroup differences (*r* = 0.659, *p* = 0.002), indicating significant differences in species evenness between the two groups. (**C-E**) Taxonomic analysis: (left) Relative abundance at phylum/genus/ species levels; (right) LDA-identified differential taxa (Firmicutes/Synergistota highlighted). (**F**) KEGG pathway heatmap revealed functional divergence (color gradient: relative abundance).

To investigate the changes in the gut microbiota of obese pregnant women, we collected fecal samples from six obese pregnant women and six normal pregnant women. After filtering and centrifugation, the fecal bacteria were prepared for 16S rRNA gene sequencing. Alpha-diversity analysis revealed a significant reduction in gut microbial diversity in the obesity pregnancy group compared to the normal pregnancy group, as evidenced by statistically significant decreases in key indices, including the abundance-based coverage estimator (ACE), Chao1 richness index, Faith's phylogenetic diversity (Faith-PD), Observed coverage, and Shannon index (Figs. 1A and S2A-S2E). Beta-diversity analysis further indicated that microbial community richness was markedly lower in the obesity group (Figs. 1B and S2F-S2J). As depicted in Fig. S1A-S1B, the distribution of the top 20 microbes at the genus level in the two groups is presented. In addition, the rank abundance curve demonstrates that the sequencing sample information has been adequately explored (Fig. S1C). The normal and obesity groups share 156 common microbes, with 31 unique to the normal group and 25 unique to the obesity group (Fig. S1D). LEfSe analysis at the phylum (Fig. 1C), genus (Fig. 1D), and species (Fig. 1E) levels confirmed that gut microbiota diversity in gestational obese women is significantly reduced across all taxonomic levels. Compositional analysis of microbial communities (Fig. 1F) revealed distinct structural differences between the two groups. Collectively, these findings establish a robust association between GO and comprehensive declines in both microbial species richness and phylogenetic diversity.

### The Gut Microbiome of Pregnant Obese Women Is associated with Pregnancy Outcome and Neonatal Index

To assess the relationship between gut microbiota composition and pregnancy outcomes/neonatal parameters in GO, the following experimental investigations were performed.

**Fig. 2:**
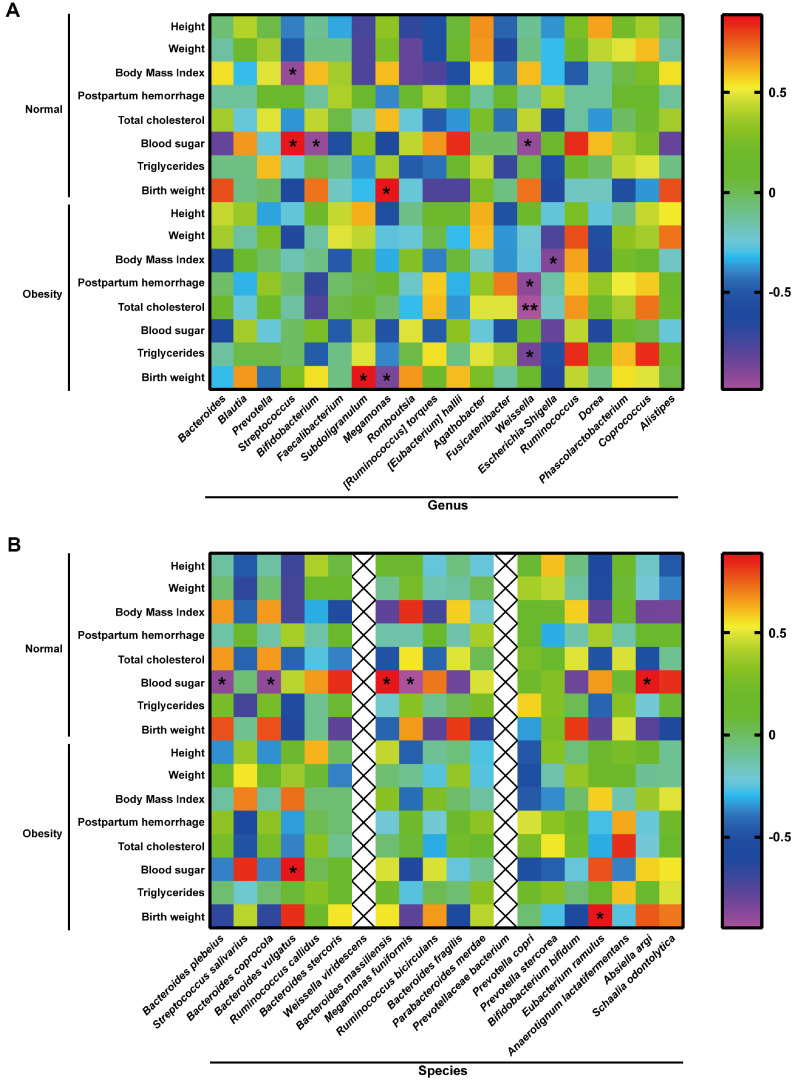
Gut microbiome of pregnant obese women is associated with pregnancy outcome and neonatal index. (**A**) Heatmaps comparing gut microbiome and pregnancy outcome at genus level in normal and obesity groups. (**B**) Heatmaps comparing gut microbiome and pregnancy outcome at the species level in normal and obesity groups. *p < 0.05, **p < 0.05.

Fig. 2A illustrated that, in the normal group, blood glucose levels were positively correlated with *Streptococcus* (*r* = 0.8857, *p* = 0.030) and negatively correlated with *Bifidobacterium* (*r* = -0.9429, *p* = 0.020) and *Weissella* (*r* = -0.9429, *p* = 0.020). Additionally, BMI showed a negative correlation with *Streptococcus* (*r* = -0.9429, *p* = 0.020), and birth weight exhibited a positive correlation with *Megamonas* (*r* = 0.8857, *p* = 0.030). In the obesity group, postpartum hemorrhage (*r* = -0.9241, *p* = 0.020), total cholesterol (*r* = -0.9856, *p* = 0.006), and triglycerides (*r* = -0.8697, *p* = 0.030) were significantly negatively correlated with *Weissella*, while BMI showed a negative correlation with *Escherichia-Shigella* (*r* = -0.8986, *p* = 0.030). Neonatal birth weight was positively correlated with *Subdoligranulum* (*r* = 0.8857, *p* = 0.030) but negatively correlated with *Megamonas* (*r* = -0.8804, *p* = 0.050). Fig. 2B revealed significant negative correlations between neonatal blood glucose and *Bacteroides plebeius* (*r* = -0.8857, *p* = 0.030), *Bacteroides coprocola* (*r* = -0.8857, *p* = 0.030), *Megamonas funiformis* (*r* = -0.9429, *p* = 0.020), and positively correlated with *Absiella argi* (*r* = 0.8804, *p* = 0.050) in the normal pregnancy group, while a positive correlation was observed with *Bacteroides massiliensis* (*r* = 0.8857, *p* = 0.030). In summary, the changes in the gut microbiome in maternal obesity are related to pregnancy outcome and neonatal index.

### LTA Intervention Ameliorated the Metabolic Parameters in Pregnant Mice Induced by FMT Combined with HFD

**Fig. 3:**
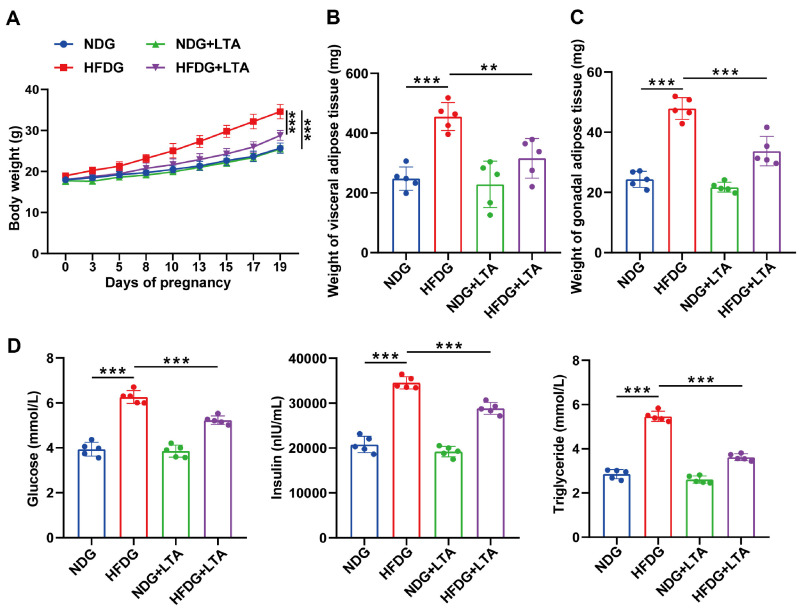
LTA intervention ameliorated metabolic parameters in pregnancy mice induced by FMT combined with HFD. (**A**) Changes in body weight throughout pregnancy were analyzed in each group. (**B**) The weight of visceral adipose tissue was measured in each group. (**C**) Weight of gonadal adipose tissue in each group. (**D**) Fasting glucose, insulin and cholesterol levels were assessed in each group. **p < 0.01, ***p < 0.001.

To further investigate whether LTA intervention impacts the metabolic parameters of pregnant mice induced by FMT combined with HFD, we conducted FMT on normal pregnant mice and HFD pregnant mice, respectively. Subsequently, LTA was administered to both groups. As depicted in Fig. 3A, in contrast to the mice in the HFDG, the mice in the HFDG+LTA group exhibited decreased body weight, suggesting that LTA can attenuate weight increase. As illustrated in Fig. 3B-3C, the mice in the HFDG were able to decrease the weight of visceral and gonadal adipose tissue following LTA treatment. The ELISA results demonstrated that LTA treatment significantly reduced the serum levels of glucose, insulin, and cholesterol in HFDG mice (Fig. 3D). In summary, LTA intervention ameliorated the metabolism of pregnant mice induced by FMT combined with HFD.

### LTA Ameliorated FMT- and HFD-Induced Intestinal Permeability in Pregnant Mice Correlated with Modulation of FGF15/FXR Activity

**Fig. 4:**
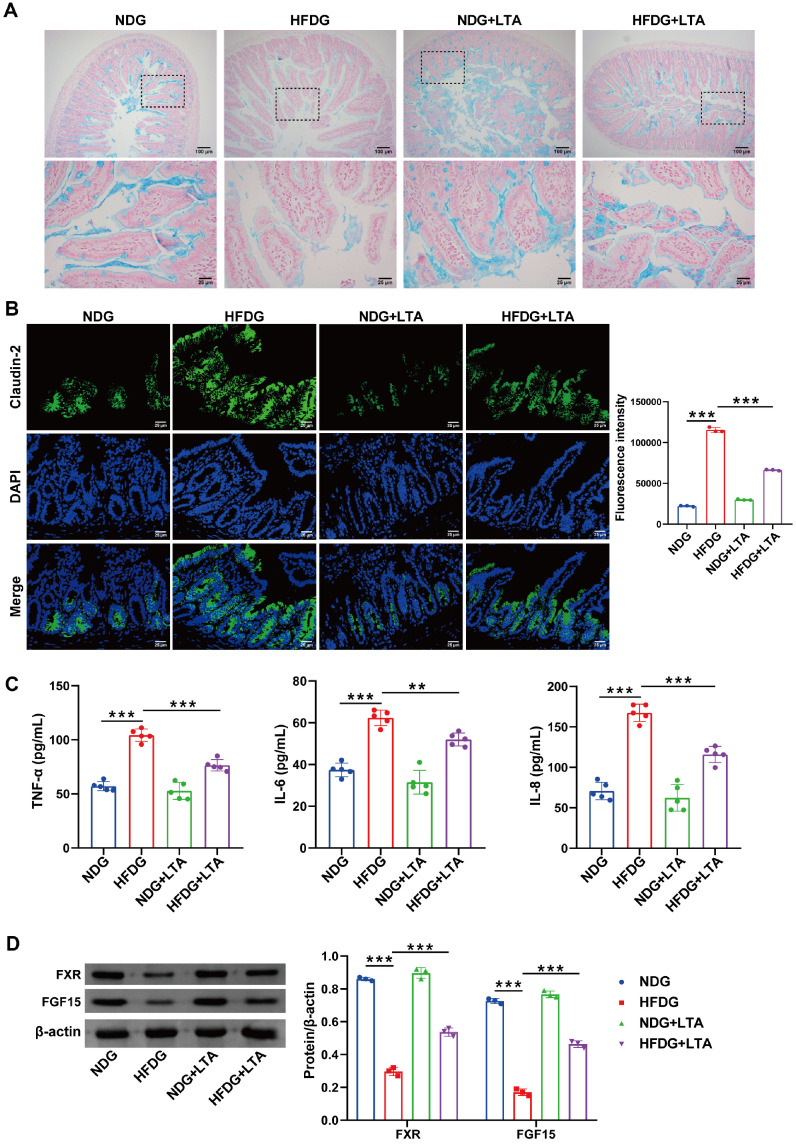
Gut microbiome of pregnant obese women is associated with pregnancy outcome and neonatal index. (**A**) The histopathology changes in intestinal villi were observed by HE staining. (**B**) The expression of the Claudin-2 protein was detected by IF (**C**) The concentration of TNF-α, IL-6 and IL-8 was detected by ELISA (**D**) The expression of FXR and FGF 15 was detected by WB. **p < 0.01, ***p < 0.001.

HE staining was employed to assess morphological changes in intestinal villi across mouse groups for evaluation of the mucosal barrier. HE staining showed that HFDG decreased intestinal mucus thickness and colonic mucosal damage, whereas LTA intervention restored mucus thickness and ameliorated mucosal damage in the HFDG+LTA group mice (Fig. 4A). Claudin-2, a member of the tight junction protein family, is crucial for regulating paracellular permeability and maintaining epithelial barrier function. However, its overexpression is linked to intestinal barrier dysfunction and heightened inflammatory responses [22, 23]. Immunofluorescence results showed that, compared to the HFDG, the expression of Claudin-2 protein was decreased in the HFDG+LTA group, indicating that LTA reduced the permeability of the mice intestine (Fig. 4B). Fig. 4C demonstrated that the levels of inflammatory factors TNF-α, IL-6, and IL-8 were lower in the HFDG+LTA group pregnant mice than in the HFDG, implying that LTA reduced inflammatory reaction. Fibroblast growth factor 15 (FGF15), a downstream target of FXR [24], collaborates with FXR to preserve intestinal barrier integrity and reduced permeability through modulation of tight junctions, anti-inflammatory effects, and mucus layer reinforcement [25]. Western blot analysis revealed increased expression of FXR and FGF15 in LTA-treated HFDG mice, suggesting that LTA may decrease intestinal permeability in pregnant mice through the upregulation of FXR and FGF15 (Fig. 4D). Collectively, the data indicate that LTA ameliorates FMT- and HFD-induced intestinal permeability in pregnant mice correlated with modulation of FGF15/FXR activity.

### LTA Attenuated Hepatic Lipid Metabolism in Pregnant Mice Induced by FMT Combined with HFD, and Has Potential Correlation with the FXR/FGF15 Pathway

**Fig. 5:**
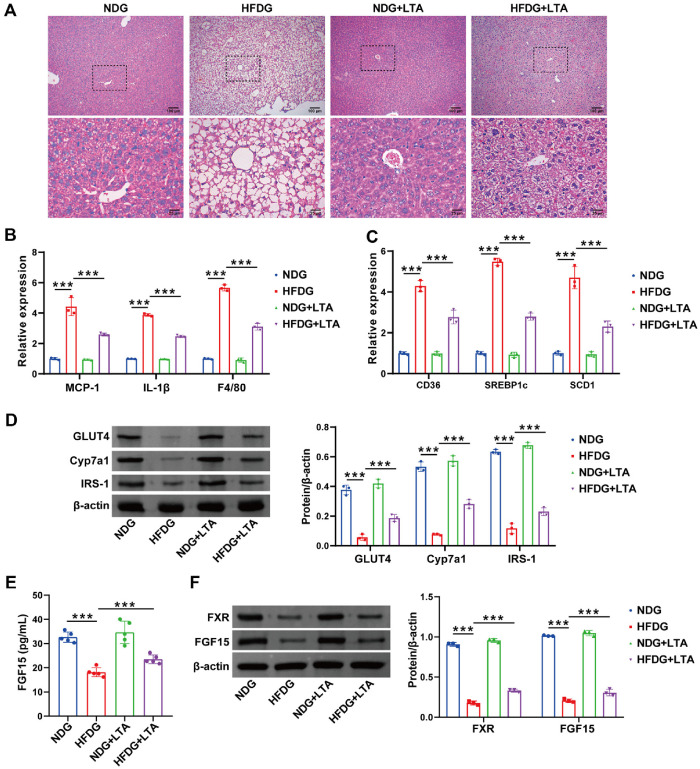
LTA modulates FXR/FGF15 to improve hepatic lipid metabolism in pregnant mice induced by FMT combined with HFD. (**A**) The histological changes in the liver were observed using HE staining. (**B**) ELISA detected the relative expression of MCP-1, IL-1β, and F4/80. (**C**) RT-qPCR detected the relative expression of CD36, SREBP1c, and SCD1. (**D**) WB observed the expression levels of GLUT4, Cyp7a1, and IRS-1. (**E**) The concentration changes in FGF15 were observed by ELISA. (**F**) The changes of FXR and FGF15 were observed by WB. ***p < 0.001.

To explore the function of LTA in modulating liver lipid metabolism through FGF15 in pregnant mice, the following experiments were performed. HE staining results showed that compared to the HFDG, mice in the HFDG+LTA group demonstrated markedly decreased liver fat deposition, suggesting the role of LTA in mitigating HFD-driven hepatic steatosis (Fig. 5A). Monocyte chemoattractant protein-1 (MCP-1), alternatively designated as C-C motif chemokine ligand 2 (CCL2), is a pivotal member of the chemokine superfamily that orchestrates innate immune responses and modulates inflammatory processes across tissues [27, 28]. As shown in Fig. 5B, the HFDG exhibited elevated hepatic levels of MCP-1, inflammatory marker IL-1β, and macrophage marker F4/80 versus the NDG, which were attenuated by LTA treatment. Subsequent assessment of hepatic lipid metabolism genes (CD36, SREBP1c, SCD1) [26] revealed that LTA significantly suppressed their expression in the HFDG (Fig. 5C). GLUT4 governs liver glucose metabolism [27], Cyp7a1 sustains cholesterol balance, and IRS-1 coordinates lipid metabolism through fatty acid synthesis stimulation and lipolysis suppression [28]. Increased protein levels of GLUT4, Cyp7a1, and IRS-1 in the HFDG+LTA group versus HFD alone were observed via western blot, highlighting the beneficial effects of LTA on liver lipid metabolism (Fig. 5D). In addition, the downregulation of FGF15 and FXR proteins in FMT+HFD pregnant mice was reversed by LTA treatment, as evidenced by elevated peripheral blood protein levels (Fig. 5E and 5F). Collectively, these data indicate improved hepatic lipid metabolism by LTA in the gestational obesity model, with observed changes in the FXR/FGF15 pathway.

### LTA Administration Ameliorated the Junctional Zone of Placental Tissue in Gestational Mice Induced by FMT Combined with HFD

**Fig. 6:**
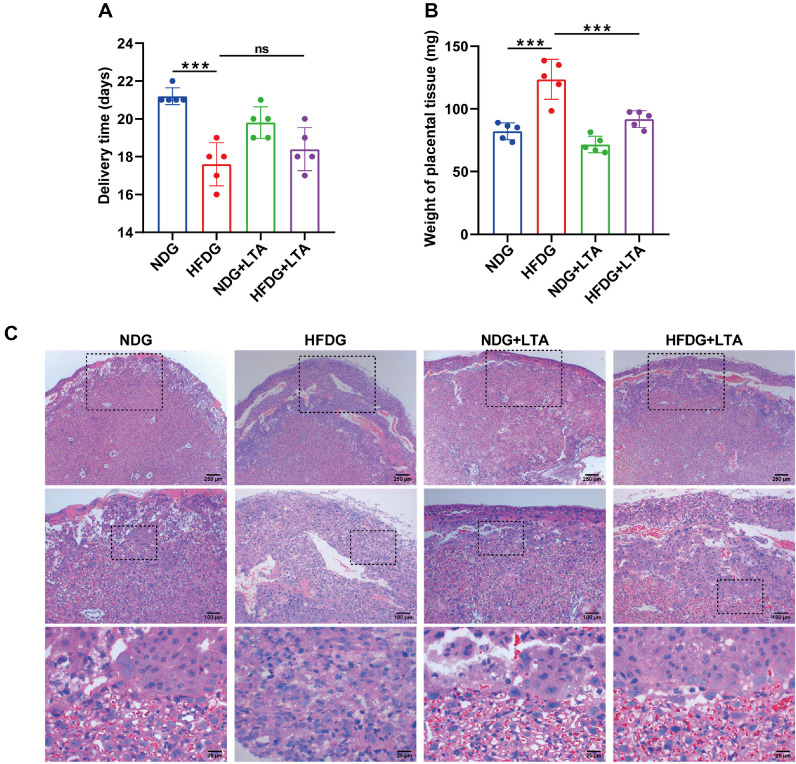
LTA administration ameliorated the junctional zone of placental tissue in gestational mice induced by FMT combined with HFD. (**A**) The changes in delivery time were assessed. (**B**) The weight of the placental tissue was measured. (**C**) The histological changes of placental tissue were detected by HE staining. ns, not significant, *p < 0.05, ***p < 0.001.

To further explore the impact of LTA on delivery outcomes in HFD combined with FMT-induced gestational mice, we performed the following experiments. As shown in Fig. 6A and 6B, while no differences in parturition timing were observed between HFDG and HFDG+LTA groups, the latter demonstrated significantly decreased placental tissue weight, indicating that LTA administration in FMT combined with HFD pregnancies may mitigate placental tissue mass. Aberrant placental layer organization could signal pathological manifestations. In rodents, the mature placental structure consists of three distinct compartments: the external maternal decidua, junction zone (JZ), and internal labyrinth zone [29]. The findings demonstrate that FMT combined with HFD induction caused substantial JZ thickening in murine placentas, which was significantly attenuated by LTA treatment. However, no intergroup differences were detected in the labyrinth or decidual layers (Fig. 6C), suggesting that LTA administration ameliorates the junctional zone of placental tissue in gestational mice induced by FMT combined with HFD.

### LTA Administration Restored the Weight of Mice Pups in Gestational Mice Induced by FMT Combined with HFD

**Fig. 7:**
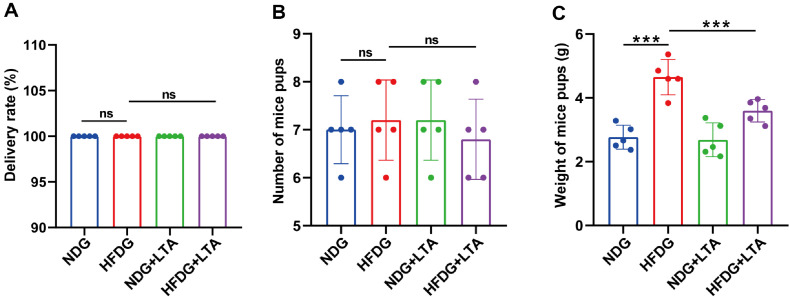
LTA administration restored the weight of mice pup in gestational mice induced by FMT combined with HFD. (**A**) The delivery rate of each group of mice was recorded. (**B**) The number of mice pups in each group was recorded. (**C**) The weight of mice pups in each group was recorded. ns: no significant, *p < 0.05, ***p < 0.001.

As shown in Fig. 7A and 7B, no significant differences were observed in parturition rates or litter size across experimental groups. However, Fig. 7C demonstrated that the HFDG mice induced by FMT combined with HFD significantly increased pup weight compared to the NDG. Notably, the HFD+LTA group exhibited reduced weight of mice pups relative to the HFDG. These findings suggest that GO exacerbates offspring weight gain, while LTA intervention effectively mitigates this metabolic alteration.

## Discussion

As a worldwide public health concern, the prevalence of GO has escalated significantly in correlation with increasing obesity levels [29]. Although current research has partially elucidated its risks, the intricate pathogenic pathways and constraints of therapeutic approaches continue to pose major obstacles in this domain. Emerging data indicated that LTA could regulate body weight via appetite suppression and metabolic adjustments [17], yet its precise mechanistic role in GO requires clarification. Our research demonstrates that LTA mitigates the pregnancy outcome in the FMT+HFD-induced gestational obesity mice, with observed changes in the FXR/FGF15 signaling axis correlating to this protective effect.

Comparative investigations in humans and animal models have revealed increased vulnerability to multiple chronic pathologies in the offspring of obese mothers [30]. Maternal weight accumulation during pregnancy shows associations with epigenetic signatures in placental tissue and latent obesity-related genes in progeny [31]. HFD consumption is linked to elevated GO rates, with gut microbiome dysbiosis and intestinal-origin lipocalin-bile acid changes constituting contributors to enteric neuropathy pathogenesis [32]. The experimental data demonstrate HFD-triggered diminishment of intestinal flora diversity and increased liver metabolic stress in pregnancy-related obesity. Additionally, HFD induces marked junctional zone thickening in mice placentas, associated with compromised gestational outcomes, aligning with previous research findings.

FMT-HFD co-administration serves as a well-established experimental paradigm for studying diverse mechanisms of disease pathogenesis [33, 34]. Da Zhou *et al*.'s research demonstrated that a combined intervention of HFD-FMT alleviated HFD-induced steatohepatitis in mice [39]. FMT alters intestinal flora-regulated bile acid (BA) metabolic processes in gestational diabetes, which are critically involved in HFD-driven disease pathogenesis [35]. This investigation demonstrates that HFD-triggered GO in mice is characterized by a marked loss of intestinal flora diversity and disturbances in lipid metabolism, showing associations with both parturition outcomes and neonatal health metrics. In FMT-HFD-established GO models, LTA administration improved metabolic abnormalities and pregnancy complications associates with FXR/FGF15 pathway alterations. The results demonstrate concordance with current scientific literature.

LTA, a non-proteogenic amino acid primarily present in tea, exhibits dual hydrophilic-lipophilic properties in its molecular configuration, facilitating blood-brain barrier permeability. Recent studies have revealed that LTA mitigates microbial imbalance by decreasing the proportion of Firmicutes/Bacteroidetes and elevating fecal SCFA concentrations while improving adiposity and liver lipidosis in HFD models [36]. The compound mediates biological benefits via microbial community restructuring and gut immunological modulation. Additionally, LTA displays disease-modifying capacity via pathway-specific targeting, including the mitigation of MPTP-triggered Parkinsonism through coordinated regulation of Wnt/β-catenin and MAPK signaling cascades [37]. Our experimental data showed LTA administration markedly enhanced metabolic profiles, gut barrier function, liver lipid homeostasis, and delivery outcomes, with concurrent restoration of the weight of mice pups. These findings not only clarify the multi-target mechanisms of LTA in GO, but also offer a theoretical basis for developing gut microbiota and lipid metabolism-based intervention strategies.

Despite the significant findings of this study, certain limitations remain. First, in this study, we selected six participants with stable obesity traits from a cohort of 28 pregnant women for comparison of gut microbiota. While the large effect-size differences observed in this subgroup provide mechanistic hypotheses, the microbiota comparison is limited by the sample size. Future multi-center studies with larger cohorts are needed to establish universality. Second, by combining FMT from clinical patients with HFD, we established a mice model to validate the role of LTA and demonstrated its beneficial effects in mice with gestational obesity. However, loss-of-function experiments for the FXR/FGF15 pathway require further investigation. Future research should focus on these aspects: on the one hand, evaluate the therapeutic effects and safety of LTA in larger-scale animal experiments and clinical cohorts. On the other hand, systematically investigate the effects of LTA on short-chain fatty acids, tryptophan metabolism, and other pathways, as well as its interaction with the FXR/FGF15 signaling pathway. Finally, conduct long-term intervention trials in pregnant women with obesity to clarify the impact of LTA on maternal and fetal health.

Our study used FMT from obese pregnant women combined with HFD to replicate both the obese phenotype and characteristic gut dysbiosis seen in gestational obesity. This dual-induction approach ensures that the effects of LTA intervention better reflect the true pathophysiology and clinical relevance. However, administering LTA to pregnant mice receiving obese FMT while on a normal diet showed no significant improvements in metabolic parameters (body weight, blood glucose, insulin, cholesterol, intestinal permeability, hepatic lipid metabolism, placental weight, or litter size). A current limitation is the absence of an "HFD-only without FMT" control group to distinguish dietary versus microbial contributions. This control group will be included in future studies.

In this study, we employed the FMT+HFD model to connect the effects of LTA to the FXR/FGF15 signaling alterations, addressing a gap in this field. Our research not only confirmed that LTA regulates gut microbiota, but also further elucidated its mechanisms in liver lipid metabolism. Additionally, LTA's ability to improve pregnancy outcomes and the weight of mice pups provides novel therapeutic approaches for GO, contrasting with previous research focused solely on maternal metabolism.

## Supplemental Materials

Supplementary data for this paper are available on-line only at http://jmb.or.kr.



## Figures and Tables

**Table 1:** Antibody information.

Name	Dilution rate	Source	Cat. number	Company	Country
FXR	1: 5000	Rabbit	25055-1-AP	Proteintech	USA
FGF15	1: 1000	Rabbit	ab319994	Abcam	UK
GLUT4	1: 2000	Mouse	66846-1-Ig	Proteintech	USA
Cyp7a1	1: 5000	Rabbit	18054-1-AP	Proteintech	USA
IRS-1	1: 20000	Rabbit	17509-1-AP	Proteintech	USA
β-actin	1: 5000	Rabbit	AWA80002	Abiowell	China

**Table 2:** Primer sequences.