# Supplementary material for: L-Theanine Ameliorates Metabolic Dysregulation and Adverse Fetal Outcomes in a Mice Model of Gestational Obesity: Association with FXR/FGF15 Signaling
Source: J Microbiol Biotechnol. 2025 Sep 22;35:e2504017. doi: 10.4014/jmb.2504.04017 (PMC12535861; doi:10.4014/jmb.2504.04017)
Supplement: Supplementary file 1 [file jmb-35-e2504017-supple.pdf]

J. Microbiol. Biotechnol. 2025. 35: e2504017

https://doi.org/10.4014/jmb.2504.04017

Supplementary Table and Figures

Table S1. Clinical information for Obesity and Normal pregnant women groups.

Variate	Obesity (n=14)	Normal (n=14)	*P*-value
Age (years)	27.5±3.898	29.43±2.821	0.1457
BMI (kg/m²)	28.88±3.354	20.62±2.586	<0.0001*
Postpartum blood loss (mL)	221.4±72.63	221.4±42.58	>0.9999
Total cholesterol（mmol/L）	5.171±1.455	6.248±0.9506	0.0285*
Fasting blood glucose (mmol/L)	6.016±3.383	4.109±0.4183	0.0461*
Hemoglobin (g/L)	125.0±12.13	120.6±8.785	0.2894
Birth weight (g)	3543±501.1	3029±491.0	0.0109*

* $P<0.05$

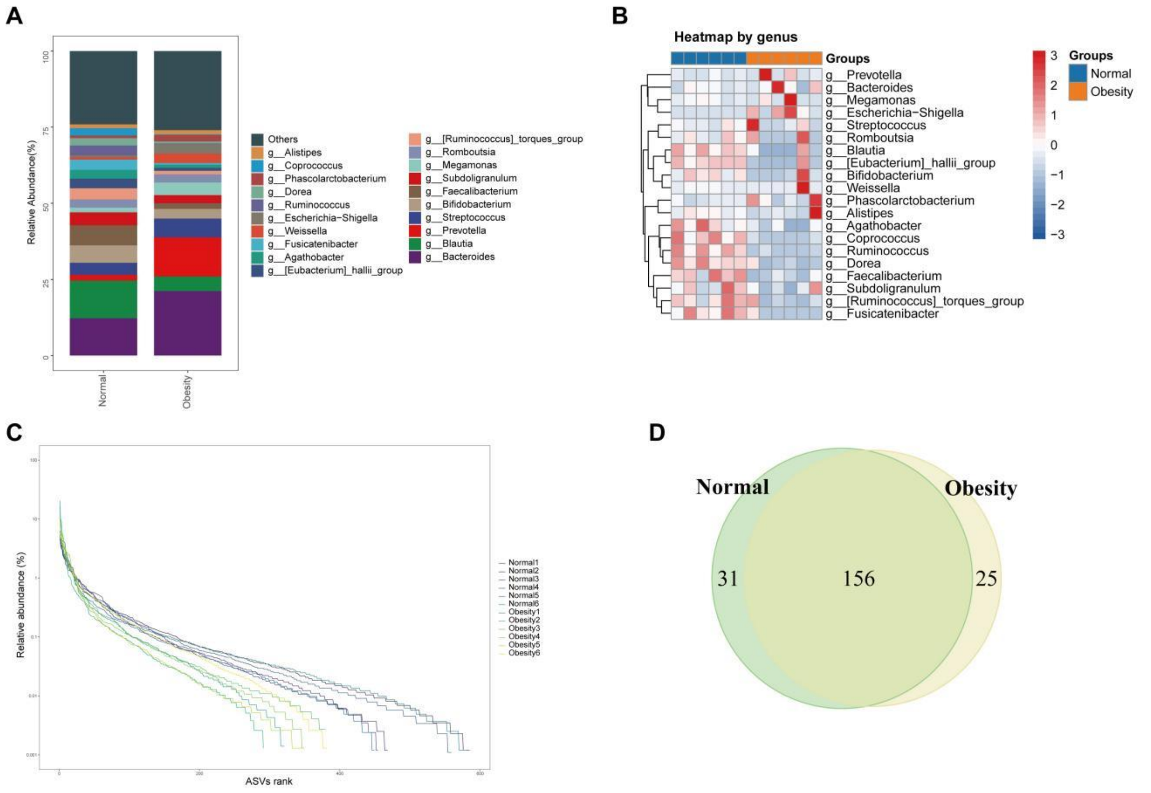

Fig. S1 A. Stacked bar chart illustrating the relative abundance of different bacterial genera in the normal and obesity groups. **B.** Heatmap depicting the abundance of bacterial genera across samples in the normal and obesity groups. **C.** Rank abundance curve showing the distribution of amplicon sequence variants (ASVs) in the normal and obesity groups. **D.** Venn diagram illustrating the shared and unique ASVs between the normal and obesity groups.

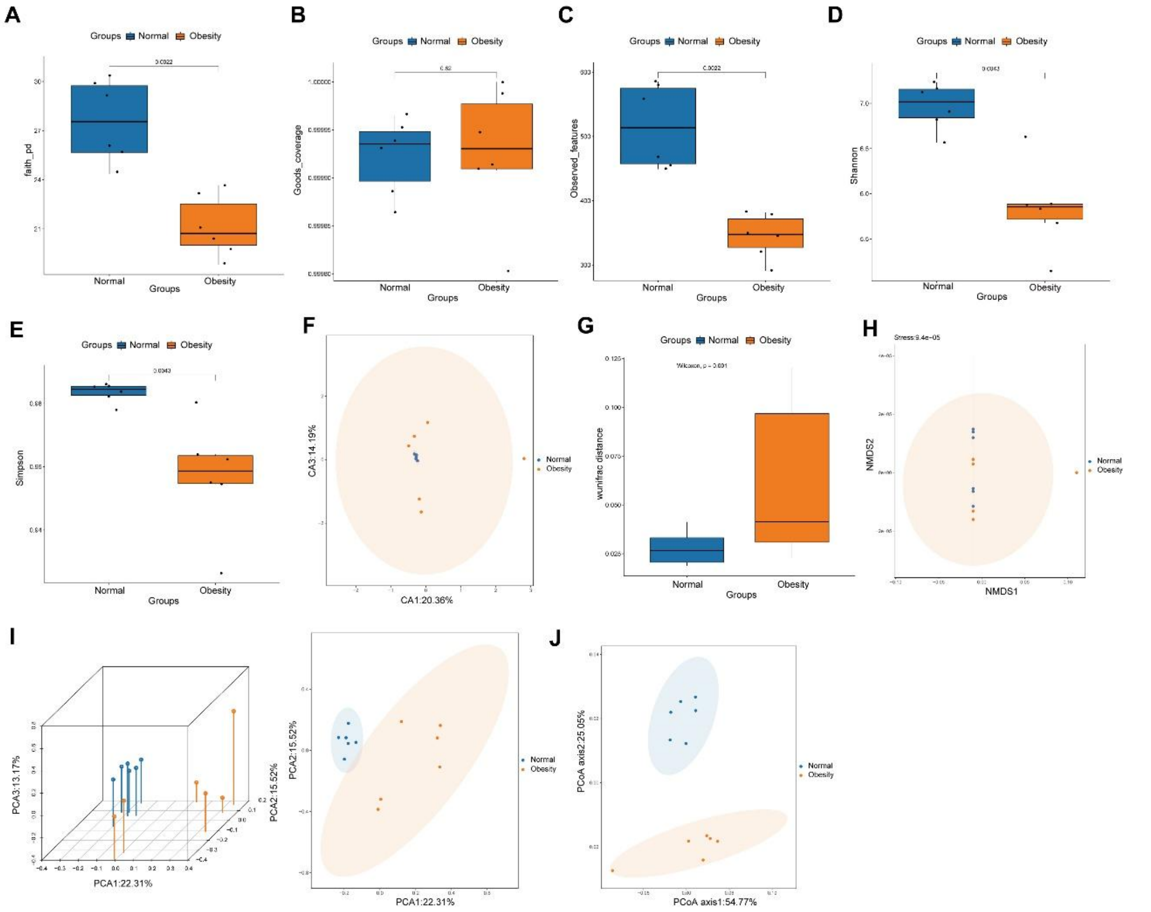

Fig. S2 (A) β-diversity analysis (Faith's Phylogenetic Diversity) revealed significantly reduced phylogenetic diversity in the obesity group versus controls (*P*=0.002), indicating greater phylogenetic diversity in the normal group. (**B)** Species coverage evaluated by Good's coverage index showed diminished values in the obesity group (*P*=0.002), indicating greater species coverage in the normal group. (**C**). Observed features analysis demonstrated decreased microbial species diversity in obese pregnancies (*P*=0.002), indicating greater species diversity in the normal group. **(D-E)** Shannon (*P*=0.002) and Simpson (*P*=0.004) indices confirmed reduced species evenness in the obesity group, indicating greater species evenness in the normal group. (**F)** Principal coordinate analysis (PCoA) (CA1:20.36%; CA2:14.19%) displayed tighter clustering in controls versus dispersed obesity samples, indicating significant differences in gut microbiota composition between the two groups. **(G)** Weighted unifrac distance analysis confirmed compositional divergence (Wilcoxon test, *P*=0.001), indicating significant differences in gut microbiota composition between the two groups. (**H)** NMDS ordination showed distinct clustering patterns between groups, indicating significant differences in gut microbiota composition between the two groups. (**I, J)** PCA (2D/3D) visualized significant β-diversity separation (normal: clustered; obesity: dispersed), indicating significant differences in gut microbiota composition between the two groups.
